# The Role of H_2_O_2_-Scavenging Enzymes (Ascorbate Peroxidase and Catalase) in the Tolerance of *Lemna minor* to Antibiotics: Implications for Phytoremediation

**DOI:** 10.3390/antiox11010151

**Published:** 2022-01-13

**Authors:** Marcelo Pedrosa Gomes, Rafael Shinji Akiyama Kitamura, Raizza Zorman Marques, Marcello Locatelli Barbato, Marcel Zámocký

**Affiliations:** 1 Laboratório de Fisiologia de Plantas sob Estresse, Departamento de Botânica, Setor de Ciências Biológicas, Universidade Federal do Paraná, Avenida Coronel Francisco H. dos Santos, 100, Centro Politécnico Jardim das Américas, C.P. 19031, Curitiba 81531-980, Brazil; rafaelkitamura@hotmail.com (R.S.A.K.); raizzazorman@gmail.com (R.Z.M.); marcello.locatelli@ufpr.br (M.L.B.); 2 Laboratory of Phylogenomic Ecology, Institute of Molecular Biology, Slovak Academy of Sciences, Dúbravská cesta 21, SK-84551 Bratislava, Slovakia; marcel.zamocky@savba.sk or; 3 Department of Chemistry, Institute of Biochemistry, University of Natural Resources and Life Sciences, Muthgasse 18, A-1190 Vienna, Austria

**Keywords:** peroxidase, catalase, amoxicillin, ciprofloxacin, erythromycin, peroxidase inhibitors

## Abstract

We investigated the individual and combined contributions of two distinct heme proteins namely, ascorbate peroxidase (APX) and catalase (CAT) on the tolerance of *Lemna minor* plants to antibiotics. For our investigation, we used specific inhibitors of these two H_2_O_2_-scavenging enzymes (*p*-aminophenol, 3-amino,1,2,4-triazole, and salicylic acid). APX activity was central for the tolerance of this aquatic plant to amoxicillin (AMX), whereas CAT activity was important for avoiding oxidative damage when exposed to ciprofloxacin (CIP). Both monitored enzymes had important roles in the tolerance of *Lemna minor* to erythromycin (ERY). The use of molecular kinetic approaches to detect and increase APX and/or CAT scavenging activities could enhance tolerance, and, therefore, improve the use of *L. minor* plants to reclaim antibiotics from water bodies.

## 1. Introduction

Environmental contamination by antibiotics has grown to be a global concern. In addition to the deleterious effects of antibiotics on the biota [1,2,3], their presence in environmental matrices contributes to the selection of resistant microbial species that constitute threats to global health [4]. The use of plants to reclaim antibiotics has emerged as a green technology in light of the inefficiency of physicochemical processes for removing antibiotics from the environment [5]. In this context, aquatic macrophytes have demonstrated positive performances in phytoremediation programs [5,6,7], although their use is still limited by their tolerance to those contaminants. The elucidation of the complex mechanisms involved in plant tolerance to antibiotics could, therefore, contribute to the identification of suitable phytoremediator species, as well as improve their phytoremediation capacities [5].

Although plants are not the primary target of antibiotics, exposure to those xenobiotics can disrupt plant metabolism, with oxidative events (mainly hydrogen peroxide (H_2_O_2_) production) frequently being observed [3,5,8]. Among H_2_O_2_-scavenging enzymes, heme containing catalase (CAT) and ascorbate peroxidase (APX) are particularly responsive in plants exposed to antibiotics when compared to heme secretory peroxidases and (non-heme) glutathione peroxidase [7]. Therefore, studies focused on APX and CAT activity in plants under antibiotic-derived stress conditions have raised particular interest [5]. *Lemna minor* exposed to amoxicillin (AMO; 2 µg L^−1^) [5] or erythromycin (ERY; 1.7 µg L^−1^) [9], *Elodea canadensis* exposed to enrofloxacin (ENR; 0.75 to 1.50 µg L^−1^) [10], and *Myrioplyllum aquaticum* and *Rotala rotundufolia* exposed to ERY (1.7 µg L^−1^) [9], for example, all showed increased catalase (CAT) and ascorbate peroxidase (APX) activities that resulted in decreased concentrations of intracellular hydrogen peroxide (H_2_O_2_) and thus prevented oxidative stress as well as the deleterious effects of those antibiotics. Increased CAT activity, with no increase in lipid peroxidation, was similarly observed in *L. minor* plants exposed to ciprofloxacin (CIP; 0.195 mg L^−1^) [11]. In contrast, the exposure of *L. minor* [8] and *Ricciocarpus natans* plants [7] to 1.05–3.05 mg CIP l^−1^ decreased their APX activity, increased H_2_O_2_ concentrations, and provoked phytotoxicity symptoms. It, therefore, appears that specific H_2_O_2_-scavenging enzymes (mainly APX and CAT) are involved in plant tolerance to antibiotics. Although exposure to AMO (0.0001 to 1 mg L^−1^) resulted in increased CAT and APX activities in the aquatic plant *Spirodela polirhiza*, phytotoxicity symptoms were still observed when it was exposed to that antibiotic [12]. Similarly, increases in peroxidase activity did not prevent reactive oxygen species (ROS; including superoxide and H_2_O_2_) accumulations and toxic effects on photosynthesis in *Vallisneria natans* plants exposed to sulfonamide (SUL; 30 and 50 mg L^−1^). Increased APX activity in *Salvinia molesta* did not prevent H_2_O_2_ accumulation, lipid peroxidation, and decreases in the relative growth rates of plants exposed to ERY (1.7 µg L^−1^) [9]. Similarly, increased CAT activity in the microalga *Raphisocelis subcaptata* likewise did not prevent the deleterious effects of sulfamethaxazole (SMX; 0.1 to 0.9 mg L^−1^) [13]. As such, the exact roles of APX and CAT in terms of their contribution to antibiotic tolerance in plants still remains unclear.

Observed antioxidant activities in plants have been linked to their capacities for reclaiming contaminants [14,15]. Thus, the elucidation of the roles of APX and CAT on plant antibiotic tolerance may provide the basis for biotechnological approaches involving plant antioxidant systems that could improve the phytoremediation capacities of aquatic macrophytes [5]. We, therefore, investigated the individual and combined roles of APX and CAT on tolerance to three antibiotics (AMX, ERY, and CIP) in the aquatic macrophytes *L. minor*—a plant species dedicated for antibiotic-phytoremediation programs—using specific inhibitors of these heme antioxidant enzymes [5,9].

## 2. Materials and Methods

### 2.1. Plant Material and Bioassays

The free-floating macrophyte *Lemna minor* L. (Araceae) was obtained from axenic cultures held at the Laboratory of Plant Stress Physiology (UFPR, Curitiba, Brazil). Before initiating the bioassays, the plants were grown in biological oxygen demand culture chambers for 25 days in aquariums (30 L) containing sterile Bold’s Basal medium (BBM) [16] (pH 7.0 ± 0.5) at 22 ± 3 °C, under a 12-h photoperiod (100 µmol photons m^−2^ s^−1^).

For the bioassays, the plants were transferred to 250-milliliter Erlenmeyer flasks (each constituting one replicate) containing 100 mL of sterile BBM with appropriate concentrations of antibiotics and/or APX and CAT inhibitors (with pH corrected to 7.0 ± 0.5, when necessary). Four flasks were used in each of the treatments (as described below). The bioassays were conducted under the same temperature and illumination conditions as the acclimatization, utilizing, with both species, a density of 9 g plant L^−1^ [9].

The following three drugs were tested to inhibit the activity of APX and/or CAT: *p*-aminophenol (*p*-AP; an APX inhibitor) [17], 3-amino,1,2,4-triazole (3-AT; known as a suicide CAT inhibitor) [18,19], and salicylic acid (SA; an inhibitor of both APX and CAT) [17]. The concentrations of the inhibitors to be used together with the antibiotics were determined in parallel experiments. For that purpose, the plants were exposed to increasing concentrations of *p*-AP (0, 1, 5, and 10 mM), 3-AT (0, 100, 200, and 300 mM), and SA (0, 500, 1000, and 2000 µM) for seven days. All chemicals were acquired from Sigma-Aldrich (São Paulo, Brazil). Stock solutions of *p*-AP (100 mM) and SA (10 mM) were prepared in ultra-pure water; 3-AT (1000 mM) was first diluted in a minimal volume of methanol and that solution was then completed with ultra-pure water until the required volume was reached. After exposure to those inhibitors, the activities of APX and CAT were determined spectrophotometrically as described below (Section 2.2). The concentration of methanol used in the highest 3-AT concentration (300 mM) did not induce significant alterations of enzyme activity or plant health (data not shown).

In order to study the roles of APX and CAT in antibiotic tolerance, the plants were exposed for seven days to AMX (0 and 2 µg L^−1^), ERY (0 and 1.7 µg L^−1^), and CIP (0 and 1.05 mg l^−1^) at environmental representative concentrations that were known to modulate APX and/or CAT activity and to induce (or not) phytotoxicity in plants [5,9]. These antibiotics are widely used for medical and veterinary purposes, being often detected in waters in concentrations up to mg L^−1^ [20,21,22]. Moreover, previous studies have related the APX and/or CAT activity with AMX, ERY, and CIP tolerance in *L. minor* [20,21]. Moreover, previous studies have related the APX and/or CAT activity with AMX, ERY, and CIP tolerance in *L. minor* plants [5,7,9]. In addition to the antibiotics alone, the plants were also exposed to combined concentrations of antibiotics and antioxidant-enzyme inhibitors (previously determined). Analytical-grade antibiotics obtained from Sigma-Aldrich (Oakville, Canada) were used in all experiments. Stock solutions of AMX (15 mg L^−1^), ERY (15 mg L^−1^), and CIP (5 mg L^−1^) were previously prepared in ultra-pure water and then diluted to achieve the respective nominal concentration used in the study.

### 2.2. Physiological Responses

Plant fresh weights were determined at the end of the seven-day experimental period by harvesting the plants, centrifuging them at 3000 rpm for 10 min at room temperature (in centrifuge tubes with small holes to remove surface water), and then weighing them [23]. The chlorophyll fluorescence of dark-acclimated (15 min) plants (3 plants/flask) was evaluated using a Pulse-Amplitude Modulated (PAM) fluorometer (model PAM-2500, Walz, Effeltrich, Germany). After measuring minimum (F0) and maximum (FM) fluorescence, saturating pulses were triggered at 20-s intervals with an actinic light (intensity of 100 µmol photons m^−2^ s^−1^) to calculate the quantum yields of photosystem II (FV/FM) [24]. H_2_O_2_ and malondialdehyde (MDA; lipid peroxidation) concentrations were determined using 0.1 g of plants, following Velikova et al. [25] and Hodges et al. [26], respectively. The specific activities of the enzymes’ SOD (EC 1.15.1.1) [27], APX (EC 1.11.1.11) [28], and CAT (EC 1.11.1.6) [29] were measured spectrophotometrically after determining total protein concentrations [30] in 0.1-g samples of plants homogenized in 1 mL of extracting buffer containing 100 mM phosphate (pH 7.8), 100 mM EDTA, 1 mM *L*-ascorbate, and 2% polyvinylpyrrolidone [15].

### 2.3. Statistical Analyses

The statistical analyses were performed using JMP 13.0 software (SAS Institute Inc., Cary, NC, USA). The results were expressed as the means of four replicates. The data were tested for normality (Shapiro–Wilk) and homoscedasticity (Bartlett), and subsequently evaluated statistically. The data were submitted to ANOVA, and the means compared using the Tukey test, at *p* < 0.05.

## 3. Results

### 3.1. Effects of Antioxidant Enzyme Inhibitors on Enzyme Activities

**Figure 1:**
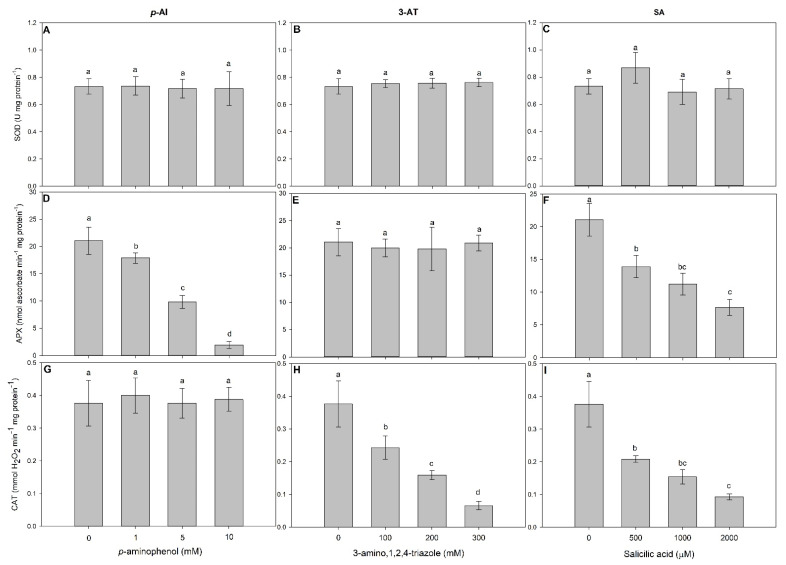
Effects of increasing concentrations of *p*-aminophenol (*p*-AI), 3-amino,1,2,4-triazole (3-AT), and salicylic acid (SA) on superoxide dismutase (SOD) (**A**–**C**), ascorbate peroxidase (APX) (**D**–**F**), and catalase (CAT) (**G**–**I**) activities in *L. minor* plants after exposure for seven days. Bars represent the means ± SD of four replicates. Different letters indicate significant differences (*p* > 0.05) using the post hoc Tukey test.

In our experiments, we have tested not only ascorbate peroxidase (APX) and catalase (CAT) but also superoxide dismutase (SOD), which is closely metabolically connected with catalase [31]. Regardless of its concentration, *p*-AI had no significant effect (*p* > 0.05) on the activities of SOD (F = 0.05) or CAT (F = 0.18), although it decreased APX activity in plants as compared to the control (F = 131.41; *p* < 0.001) (Figure 1A,D,G). 3-AT did not significantly affect (*p* > 0.05) the activities of SOD (F = 0.46) or APX (F = 0.22), although it decreased CAT activity as compared to the control (F = 48.23; *p* < 0.001) (Figure 1B,E,H). While SOD was not significantly affected (F = 0.38; *p* > 0.05), the SA-exposed plants showed decreased APX (F = 38.25) and CAT activities as compared to the control (F = 42.75; *p* < 0.001) (Figure 1C,F,I).

### 3.2. The Isolated and Combined Effects of Antibiotics and Antioxidant-Enzyme Inhibitors on Plant Physiology

The effects of antioxidant-enzyme inhibitors alone were studied by exposing plants to 5 mM *p*-AI, 200 mM 3-AT, or 1000 µM SA. Those inhibitor concentrations resulted in a 53% inhibition of APX, a 59% inhibition of CAT, and 46 and 58% inhibitions of APX and CAT, respectively, in relation to the control (Figure 1). Concentrations higher than those would induce severe toxicity symptoms in plants (data not shown) and were, therefore, not selected to be used in further studies.

**Figure 2:**
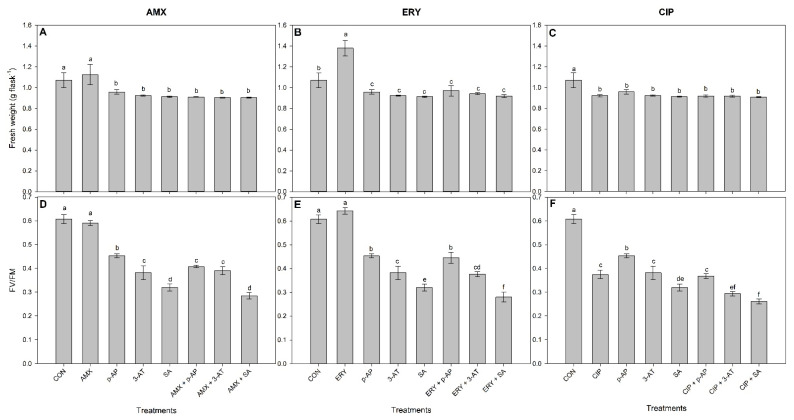
Fresh weight (**A**–**C**) and quantum yields of photosystem II (FV/FM; **D**–**F**) in *L. minor* plants exposed to isolated or combined concentrations of antibiotics and antioxidant-enzyme inhibitors for seven days. Bars represent the means ± SD of four replicates. Different letters indicate significant differences (*p* > 0.05) using the post hoc Tukey test. CON = control; AMX = 2 µg amoxicillin L^−1^; ERY = 1.7 µg erythromycin L^−1^; CIP = 1.05 mg ciprofloxacin L^−1^; *p*-AI = 5 mM *p*-aminophenol; 3-AT = 200 mM 3-amino,1,2,4-triazole; and SA = 1000 µM salicylic acid.

**Figure 3:**
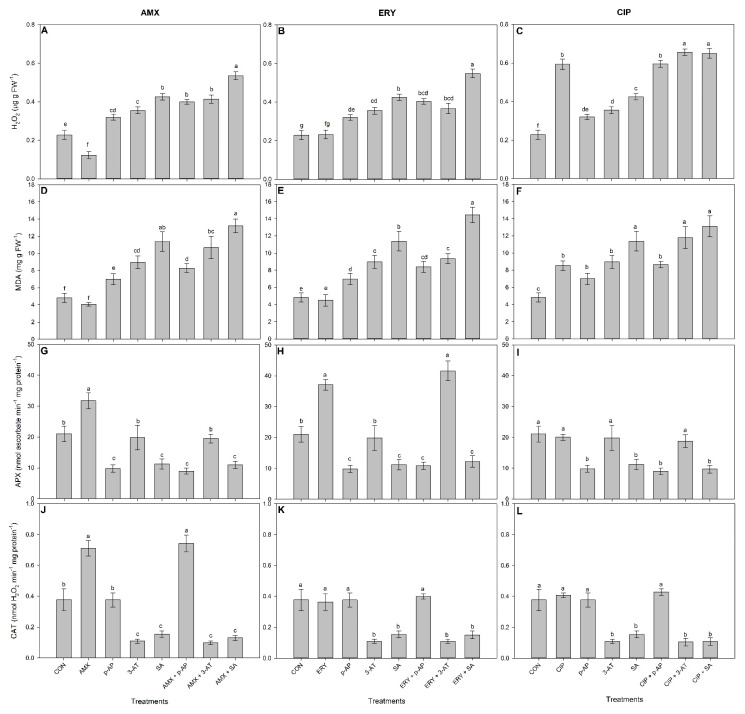
Hydrogen peroxide (H_2_O_2_; **A**–**C**) and malondialdehyde (MDA; **D**–**F**) concentrations and ascorbate peroxidase (APX; **G**–**I**) and catalase (CAT; **J**–**L**) activities in *L. minor* plants exposed to isolated or combined concentrations of antibiotics and antioxidant-enzyme inhibitors for seven days. Bars represent the means ± SD of four replicates. Different letters indicate significant differences (*p* > 0.05) using the post hoc Tukey test. CON = control; AMX = 2 µg amoxicillin L^−1^; ERY = 1.7 µg erythromycin L^−1^; CIP = 1.05 mg ciprofloxacin L^−1^; *p*-AI = 5 mM *p*-aminophenol; 3-AT = 200 mM 3-amino,1,2,4-triazole; and SA = 1000 µM salicylic acid.

While AMX alone did not induce reductions in growth (fresh weight) or photosynthesis (as evaluated by FV/FM) (*p* > 0.05), when plants were exposed to enzyme inhibitors alone or in combination with AMX, their growth (F = 15.82) and FV/FM (F = 138.25) became significantly reduced when compared to the control (*p* < 0.0001; Figure 2A,D). The plants exposed to AMX+*p*-AI showed decreased FV/FM in relation to those exposed to *p*-AI alone (Figure 2D). The H_2_O_2_ concentrations decreased in the plants exposed to AMX alone and increased in the plants exposed to inhibitors alone or in combination with AMX in relation to the control (F = 177.45, *p* < 0.0001; Figure 3A). The plants exposed to combinations of AMX with *p*-AI (AMX+*p*-AI), 3-AT (AMX+3-AT), or SA (AMX+SA) evidenced high concentrations of H_2_O_2_ in relation to those exposed only to the respective inhibitors (*p*-AI, 3-AT, or SA, respectively) (Figure 3A). The MDA concentrations in the plants were not significantly affected by AMX alone (*p* > 0.05) but increased in the plants exposed to combinations of AMX with each of the three inhibitors as compared to the control (F = 53.42; *p* = 0.0001; Figure 3D). The MDA concentrations were greater in the plants exposed to AMX+*p*-AI in relation to those exposed only to *p*-AI (Figure 3D). APX activity significantly increased in the plants exposed only to AMX and became decreased by exposure to inhibitors (with the exception of plants exposed to 3-AT) regardless of the presence of AMX in relation to the control (F = 55.64; *p* < 0.0001; Figure 3G). CAT activity was also greater in the plants exposed only to AMX but became reduced in the plants exposed to 3-AT and SA as compared to the control, regardless of the presence of AMX (F = 150.76; *p* < 0.0001; Figure 3J). The AMX+*p*-AI exposed plants showed greater CAT activity than the control (Figure 3J).

ERY alone increased the plant fresh weights (F = 57.09; *p* < 0.0001) and did not significantly affect FV/FM (F = 120.08; *p* > 0.05) in relation to the control (Figure 2B,E). The fresh weight and FV/FM significantly decreased (*p <* 0.0001), however, as compared to the control in the plants exposed to ERY in combination with all of the enzyme inhibitors (Figure 2B). FV/FM was greater in the plants exposed only to SA in relation to those exposed to ERY+SA (Figure 2E). ERY alone did not significantly affect (*p <* 0.05) the plant concentrations of H_2_O_2_ (F = 60.11) or MDA (F = 56.58) nor CAT activity (F = 70.21) (Figure 3B,E). The plants exposed to ERY with *p*-AI (ERY+*p*-AI) or SA (ERY+SA) evidenced greater concentrations of H_2_O_2_ in relation to those exposed to *p*-AI or SA alone (*p* < 0.0001; Figure 3B). The MDA concentrations were greater in the plants exposed to ERY+SA in relation to those only exposed to SA (*p* < 0.001; Figure 3D). APX activity significantly increased in the plants exposed to ERY alone as compared to the control (F = 115.32; *p* < 0.001), and that activity was greater in the plants exposed to ERY+3-AT than in those only exposed to 3-AT (Figure 3H).

Decreased fresh weight (F = 15.76) and FV/FM (F = 106.28) were observed in plants exposed to CIP and to the combination of CIP plus enzymatic inhibitors in relation to the control (Figure 2C,F). The plants exposed to the combination of CIP with *p*-AI (ERY+*p*-AI), 3-AT (ERY+3-AT), or SA (CIP+SA) showed lower FV/FM values in relation to the plants exposed to the respective inhibitor alone (*p*-AI, 3-AT, or SA) (*p* < 0.0001; Figure 2F). While the H_2_O_2_ (F = 298.48) and MDA concentrations (F = 33.65) were greater in the plants exposed only to CIP, their APX (F = 28.05) and CAT (F = 70.21) activities were not significantly affected in relation to the control (Figure 3C,F,I,L). The H_2_O_2_ and MDA concentrations were greater in the plants exposed to combinations of CIP with enzyme inhibitors as compared to the control (Figure 3C,F). Moreover, the H_2_O_2_ concentrations were greater in the plants exposed to combinations of CIP with inhibitors (CIP+*p*-AI, CIP+3-AT, and CIP+SA) in relation to those exposed to the enzyme inhibitors alone (*p*-AI, 3-AT, and SA; Figure 3C). Similarly, the MDA concentrations and CAT activities were greater in the plants exposed to CIP+3-AT than to 3-AT alone (Figure 3F,L).

## 4. Discussion

Increased productions of ROS, such as singlet oxygen (^1^O_2_), superoxide (O_2_^−^), H_2_O_2_, and hydroxyl radical (OH^.^) are typically observed in plants subjected to unfavorable conditions, such as exposure to contaminated soil and especially to antibiotics [3]. At certain levels, ROS function as signaling molecules, activating acclimatory/protective responses through transduction pathways [32]; excessive ROS concentrations, however, induce harmful effects in plant cells [33]. Defenses against ROS overproduction are activated under those circumstances, and oxidative bursts of ROS are controlled through antioxidant activities. The involvement of H_2_O_2_-scavenging enzymes, namely, of APX and CAT, have been identified as central to the plant responses to being exposed to antibiotics [3,5,11]. We confirmed here, for the first time, the importance of both antioxidant enzymes in antibiotic tolerance, and discussed the direct implications for phytoremediation technologies.

Ascorbate peroxidase and catalase both use heme prosthetic group for the catalyzed reactions but they are quite different to each other with respect to the specific reaction mechanism. Whereas ascorbate peroxidase (E.C. 1.11.1.11) can reduce hydrogen peroxide with concomitant oxidation of ascorbate, typical catalase (E.C. 1.11.1.6) can both reduce and oxidize molecules of hydrogen peroxide by releasing molecular oxygen. Thus, catalase under physiological conditions does not need any additional electron donor for recycling [31]. We have seen these differences in the results obtained for *L. minor*, with the effect of antioxidant-enzyme inhibitors [17,34]; some of them acting in a suicide inhibitory way [35]. In both cases, the active center with the prosthetic heme group is the target for suicide inhibition, although the mechanism is different [18,19,35,36] In rare cases such as SA, it was found that this inhibitor can, after binding, be (per)oxidated with catalase [17,37]. At their highest concentrations, the inhibitors induced several phytotoxic symptoms in the studied plants such as chlorosis, growth reductions, and death (data not shown)—which must be related to overaccumulations of ROS and unregulated oxidative bursts (Figure 1). At lower APX and CAT concentrations, plant growth was negatively impacted (Figure 2). These effects were also observed on photosynthetic rates. FV/FM is a proxy of photosystem II (PSII) integrity, and is highly sensitive to ROS [38]. The increased H_2_O_2_ concentrations observed in plants treated with inhibitors (Figure 3) were probably due to their interferences with the antioxidant capacities of the plants. Once accumulated, ROS suppress the de novo synthesis of the PSII D1 protein [39] and thus promotes the destruction of chloroplast membrane systems through lipid peroxidation [40] (observed in plants treated with enzyme inhibitors—Figure 3D) and the FV/FM will decrease [41]—and plant growth will be impacted.

The increased activities of antioxidant heme enzymes monitored in this study (Figure 2) apparently have the capacity to prevent the oxidative burst induced by antibiotics [5,9]. This is connected with the not observed accumulation of reactive H_2_O_2_ or MDA in plants exposed to AMX or ERY (Figure 3). The plants did, in contrast, show sensitivity to CIP alone, with decreased growth and FV/FM (Figure 2C,F). This can be caused by CIP specifically targeting the mitochondrial electron transport chain. As a result, ascorbate biosynthesis is disrupted, depriving APX of its substrate—which may explain the lack of increased APX activity in this case [42]. Moreover, under high ROS concentrations (Figure 3C), both CAT and APX are prone to protein carbonylation [8] that affects both enzyme activities. The absence of APX and CAT activities in plants exposed to CIP indicates their susceptibility to the deleterious effects of this particular antibiotic.

The inhibitory effects of applied suicide enzyme inhibitors can give valuable hints for the specific roles of two antioxidant enzymes in particular growth conditions that were the focus of our investigations. Our results clearly indicate that AMX induces ROS production and that APX is important for avoiding H_2_O_2_ accumulation and related oxidative damage (including adverse effect on the photosynthesis—c.f. lower FV/FM ratio) in the plants exposed to it. Similarly, the CAT inhibition observed in plants treated with AMX also resulted in higher H_2_O_2_ concentrations than those observed in plants treated only with the suicide inhibitor of catalase alone. Interestingly, CAT inhibition in AMX-treated plants did not result in increased oxidative damage. We, therefore, conclude that APX is more important than CAT for avoiding the damage to plants specifically caused by AMX. This assumption is reinforced by the fact that plants treated only with SA (unspecific inhibitor) showed MDA concentrations and FV/FM similar to the plants treated with AMX+SA. The expressions of different APX isoforms in distinct subcellular compartments (chloroplasts, mitochondria, peroxisome, and cytosol) are regulated in response to biotic and abiotic stress and evolved to protect plant cells against adverse conditions [42,43,44,45]. CAT activity alone was insufficient to avoid serious oxidative damage—this underlines the essential role of APX in the response to AMX exposure.

However, catalase is essential in other conditions, namely, the exposure to CIP. In the special case when CAT was inhibited by a specific inhibitor 3-AT in the CIP-treated plants, decreased FV/FM and increased H_2_O_2_ concentrations were observed (Figure 2 and Figure 3). Apparently, the presented results indicate the essential role of CAT in avoiding oxidative damage in plants selectively exposed to CIP. This antibiotic is thought to interfere with the energy transfer from excited chlorophyll in the antenna complex to the RC-II reaction center, therefore disrupting the chloroplast electron transport chain and leading to delays in the kinetics of photoreduction of the primary quinone acceptor [46], and heme catalase can protect plant photosystems from such damages.

Here, the presented results indicate that both APX and CAT activities are important for avoiding H_2_O_2_ accumulations and related oxidative damages (e.g., lipid peroxidation) in plants exposed to ERY (Figure 1E and Figure 2E). When both enzymes were inhibited by SA in the plants treated with ERY, both H_2_O_2_ and MDA concentrations increased, and FV/FM decreased in relation to the plants only treated with SA. It is plausible that some synergistic effects of APX and CAT occur in avoiding ROS damage in the plants exposed to ERY.

The capacities of various plants for reclaiming contaminants, and their phytoremediation abilities, have been directly linked to their antioxidant activities [14,15]. Investigating the specific roles of main antioxidant enzymes, namely, APX and CAT, in the antibiotic tolerance of phytoremediator species can arise opportunities for novel biotechnological approaches that could improve their capacity to reclaim drugs from contaminated water bodies. The usage of highly specific substances that would stimulate antioxidant enzyme production and increase the tolerance (and survival) of plants and their capacity to take up antibiotics from growth media is very promising. It is important, however, to stress that antibiotics occur mixed in water bodies, and it will, therefore, also be important to investigate these specific conditions. In this respect, the increased expression and activity of both APX and CAT identified in *L. minor* may positively enhance their use in programs directed towards reclaiming antibiotics from contaminated water environments.

## 5. Conclusions

Here, we demonstrated the divergent roles of the main H_2_O_2_-scavenging enzymes, APX (heme peroxidase) and CAT (heme catalase), in the acquired tolerance of *L. minor* plants to different antibiotics. Our results indicate the increased importance of APX in contrast to CAT in avoiding oxidative damage in plants exposed to AMX. CAT, however, was central to ameliorating the deleterious effects of CIP on plants, while both APX and CAT functioned together for ERY tolerance. In addition to establishing a protocol for studying the complementary roles of APX and CAT in plants using enzyme inhibitors, we confirmed the importance of these antioxidant enzymes in antibiotic tolerance, opening the possibility of using specific molecular approaches to enhance the capacity of *L. minor* plants to reclaim antibiotics from water bodies.

## Data Availability

Data are contained within the article.
